# Aneuploidy and chromosomal instability in cancer: a jackpot to chaos

**DOI:** 10.1186/s13008-015-0009-7

**Published:** 2015-05-20

**Authors:** Maybelline Giam, Giulia Rancati

**Affiliations:** Institute for Medical Biology (IMB), Agency for Science, Technology and Research (A*STAR), Singapore, 138648 Singapore; School of Biological Sciences, Nanyang Technological University, Singapore, 637551 Singapore; Department of Biochemistry, Yong Loo Lin School of Medicine, NUS, Singapore, 117597 Singapore

**Keywords:** Aneuploidy, Chromosome instability, DNA damage, Cancer evolution, Oncogene, Tumor suppressor

## Abstract

Genomic instability (GIN) is a hallmark of cancer cells that facilitates the acquisition of mutations conferring aggressive or drug-resistant phenotypes during cancer evolution. Chromosomal instability (CIN) is a form of GIN that involves frequent cytogenetic changes leading to changes in chromosome copy number (aneuploidy). While both CIN and aneuploidy are common characteristics of cancer cells, their roles in tumor initiation and progression are unclear. On the one hand, CIN and aneuploidy are known to provide genetic variation to allow cells to adapt in changing environments such as nutrient fluctuations and hypoxia. Patients with constitutive aneuploidies are more susceptible to certain types of cancers, suggesting that changes in chromosome copy number could positively contribute to cancer evolution. On the other hand, chromosomal imbalances have been observed to have detrimental effects on cellular fitness and might trigger cell cycle arrest or apoptosis. Furthermore, mouse models for CIN have led to conflicting results. Taken together these findings suggest that the relationship between CIN, aneuploidy and cancer is more complex than what was previously anticipated. Here we review what is known about this complex ménage à trois, discuss recent evidence suggesting that aneuploidy, CIN and GIN together promote a vicious cycle of genome chaos. Lastly, we propose a working hypothesis to reconcile the conflicting observations regarding the role of aneuploidy and CIN in tumorigenesis.

## Genomic instability: an engine fueling cancer progression

Cancer is a multi-stage somatic evolutionary process, where cells that have acquired mutations conferring beneficial phenotypic traits, such as sustained proliferative signaling or resistance to cell death, clonally expand and outcompete less fit neighboring cells [1–3]. Cancer cells are notoriously known for their aberrant and complex genomes and for their large cell-to-cell variation. The genomic diversity present in cancer cells ranges from single nucleotide changes to large-scale cytogenetic alterations and is caused by increased genomic instability (GIN) [4–8]. GIN, a cellular state characterized by an increased frequency of accumulating genetic alterations, is a consequence of mutations affecting pathways regulating: 1) DNA replication fidelity in S phase (including telomere maintenance), 2) cell cycle progression and checkpoint control, 3) proper chromosome segregation in mitosis, and 4) repair of sporadic DNA damage [9]. GIN has been described as an enabling characteristic of cancer cells as it increases the chances of acquiring beneficial mutations, thus enabling the acquisition of other cancer hallmarks [8, 10–12]. Importantly, GIN also increases cell-to-cell variation, leading to accumulation of standing genetic variation that could facilitate the adaptation of cancer cell populations to harsh and fluctuating milieus typical of the tumor microenvironment [13, 14]. In accordance with this view, genomic instability has been correlated with tumor progression and is associated with poor prognosis for certain types of cancer [5, 14–16]. Among the many proteins counteracting GIN by ensuring genome surveillance and maintenance is the tumor suppressor p53, nicknamed the ‘Guardian of the Genome’ [17]. p53 critically determines the fate of cells experiencing DNA damage, activating cell cycle arrest, senescence or apoptosis depending on the severity of the insult [18]. Loss of p53, though occurring at different stages and sometimes relatively late in the development of some tumors [19, 20], could remove a major block of genetic instability and allow cancer cells to accumulate further oncogenic mutations in order to progress towards increased aggressiveness [21–23].

There are two main classes of GIN: nucleotide instability and chromosomal instability (CIN) [5]. While nucleotide mutations include base substitutions, deletions and insertions, mutations at the cytogenetic level include gains and losses of whole or parts of chromosomes as well as simple or complex chromosomal rearrangements. Since the link between mutations increasing nucleotide instability and cancer predisposition has been well established [5], in this review we will focus on the more controversial role of CIN and its ‘by-product’ aneuploidy in cellular transformation and tumor progression.

## Chromosomal instability and aneuploidy: friends or foes of cellular transformation?

**Fig. 1:**
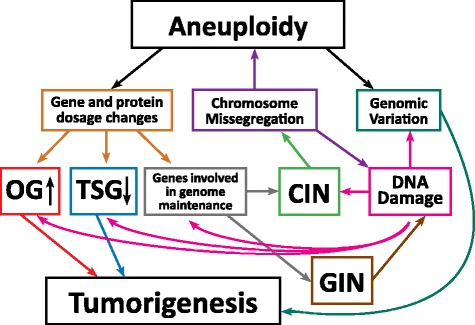
Aneuploidy, CIN and GIN loop together to tumorigenesis. Aneuploidy results in direct changes in mRNA and protein expression levels of genes found on the aneuploid chromosome. Increasing or decreasing the dosage of oncogenes (OG) and tumor suppressor genes (TSG) can have direct effects on cellular transformation. Additionally, while CIN leads to aneuploidy via increased chromosome missegregation, aneuploidy can lead to CIN by changing the stoichiometry of protein complexes required for genome maintenance or by scaling defects brought about by the presence of extra DNA. At the same time, chromosome missegregation has the potential to increase DNA damage and GIN. CIN and GIN are considered mutator phenotypes that could potentially enhance the chance of accumulating oncogenic mutations, thus promoting tumorigenesis. Their ‘by-products’, aneuploidy and DNA damage generate genetic variation, allowing cells to have increased adaptive potential in the tumor microenvironment

Chromosomal instability (CIN) refers to an increased rate of chromosome missegregation due to errors in mitosis [24, 25]. One of the main products of CIN is aneuploidy, a condition associated with the gain or loss of whole chromosomes or parts thereof leading to genomic imbalances (Fig. 1). There are many roads leading to CIN: multipolar spindles, improper chromosome condensation or cohesion, inefficient chromosome congression, defects in mitotic spindle assembly/dynamics, defective mitotic checkpoint and telomere attrition, replication stress, and improper kinetochore-microtubules attachments [25–27]. To add even more complexity, recent studies proposed that aneuploidy itself could lead to CIN (Fig. 1 and discussed below), suggesting the presence of a positive feedback loop resulting in increasing levels of aneuploidy.

As with genomic instability, CIN has been suggested to provide phenotypic variation and increase tumor heterogeneity, therefore fuelling the ability of cancer cells to progress, adapt to chemotherapy or to relapse [28]. Accordingly, in a large proportion of tumors, CIN occurs at early stages and it has been associated with poor prognosis and increased aggressiveness in multiple types of human cancer [29]. Moreover, CIN has been shown to drive metastasis after the shut-off of the oncogenic stimulus in a mouse model of KRAS-induced lung cancer [30], suggesting that the genomic changes induced by CIN are able to sustain cancer evolution upon oncogene withdrawal. Chromosomally unstable cancer cells also exhibit increased intrinsic multidrug resistance when compared to their stable counterparts [14, 31, 32]. However, the relationship between CIN and drug resistance is far from simple. Indeed, by stratifying tumors using a CIN expression signature, Swanton and colleagues found that breast tumors with the lowest or the highest CIN signatures were associated with improved prognosis relative to those with intermediate scores [15]. The same trend was also observed in ovarian, gastric and non-small cell lung cancer [15], suggesting the existence of an intermediate “sweet spot” for CIN to induce aggressive cancer behavior (see below for further discussion on this point).

A major question remains whether CIN is sufficient to initiate tumorigenesis? Indications supporting this hypothesis come from analysis of human patients with mosaic variegated aneuploidy syndrome (MVA). The MVA syndrome has been mapped to mutations in either the SAC protein BUBR1 or in the centrosomal protein CEP57 and is characterized by increased CIN [33, 34]. MVA patients are mosaic for different karyotypes and on top of developmental defects they are predisposed to childhood cancer [33–36], supporting the idea that CIN could lead to cancer in humans.

However, most of what we know in this regard comes from mouse models, many of which focus on partial inactivation of proteins which are either directly involved in the spindle assembly checkpoint (SAC), part of the signaling downstream of the SAC (e.g. securin or APC co-factors) or required for proper chromosome alignment (e.g. CENP-E) (summarized in Table 1 and reviewed in [37, 38]). While CENP-E is a motor protein required for stable spindle microtubule capture at kinetochores [39], the SAC is a conserved surveillance signaling cascade that inhibits anaphase onset in response to mis-attached chromosomes [40, 41]. A weakened CENP-E or SAC results either in increased chromosome mis-alignments or in an inability to resolve them, thus leading to chromosome missegregation and subsequent formation of aneuploidy. While homozygous knockouts of CENP-E or of any SAC signaling genes (including MAD1, MAD2, MPS1, BUB1, BUBR1, BUB3, the Bub3-related protein RAE1, and the APC cofactors CDC20 and FZR1) result in massive chromosome segregation defects and early embryonic lethality, heterozygous mice are born viable and display no overt phenotypes [37, 38, 42, 43]. Consistent with the hypothesis that CIN is sufficient to initiate cancer, heterozygous offspring of Mad1, Mad2 and CENP-E knock-out mice, as well as offspring of a Bub1 hypomorphic mouse strain showed increased incidence of spontaneous tumors mainly in the lung and the hematopoietic system (Table 1) [44–47]. Some mouse strains did not show increased spontaneous tumor formation but instead exhibited increased tumor onset when challenged with chemical carcinogens such as dimethylbenzanthracene (DMBA) or azoxymethane (AOM) (Table 1). In contrast, haploinsufficiency of Bub3 or Rae1 did not result in increased tumorigenesis [48, 49], and some mouse models even showed decreased tumor formation when challenged with carcinogens, suggesting that the relationship between impaired SAC signaling, aneuploidy and tumor onset is complex. Consistently, while overexpression of some SAC genes such as MAD2 and BUB1 has been shown to induce CIN and cancer onset, overexpression of BUBR1 has protective effects on spontaneous tumor development and accumulation of aneuploidy (Table 1) [50–52].

**Table 1:** Cancer phenotypes of CIN mouse models

Gene	Mitotic Function	Cancer phenotype of resulting mice				References
		Genotype	Spontaneous tumors	Chemically-induced tumors	Crossed with tumor-prone backgrounds	
Bub1	SAC	+/−	Not observed	DMBA-induced ()	p53^+/−^ and p53^−/−^ (=); Apc^Min/+^ (colon )	[47, 125, 127]
		H/H, H/− (*)	Tumors in various tissues in ~50 % of 20 months-old mice	NT	p53^+/−^ and p53^−/−^ (); Apc^Min/+^ (colon ); Rb^+/−^ (=); Pten^+/−^ ()	[47, 125, 127]
		overexp.	Tumors in various tissues in 60-70 % of 12–16 months-old mice	NT	NT	[51]
Mad1	SAC	+/−	Lung tumors in 19 % of 18 months-old mice	Vinicristine-induced ()	p53^+/−^ (=)	[44, 126]
Mad2	SAC	+/−	Lung tumors in 30 % of 18 months-old mice	NT	p53^+/−^ ()	[45, 126]
		overexp.	Tumors in various tissues in 50 % of 12–20 months-old mice	NT	Eu-Myc (); KRAS^G12D^ ()	[30, 52]
Mad1; Mad2	SAC	+/−; +/−	NT	NT	p53^+/−^ ()	[126]
BubR1	SAC	+/−	Not observed	AOM-induced ()	Apc^Min/+^ (colon small intestine)	[53, 57, 122]
		H/H(*)	Not observed	DMBA-induced ()	p53^−/−^ (incidence but shortened osteosarcoma latency and accelerated aging onset)	[57, 134, 135]
		overexp.	Decreased	DMBA-induced ()	KRAS^G12D^ ()	[50]
Bub3	SAC	+/−	Not observed	DMBA-induced (=)	p53^+/−^ (=); Rb1^+/−^ (=)	[48, 49]
Rae1	SAC	+/−	Not observed	DMBA-induced (=)	NT	[48]
Bub3; Rae1	SAC	+/−; +/−	Not observed	DMBA-induced ()	NT	[48]
Mps1	SAC	DK/DK (**)	Not observed	NT	p53^fl/+^ Lck-Cre + (T-ALL)	[128]
		DK/fl (**)	NT	NT	p53^fl/fl^ Lck-Cre + (=)	[128]
CENP-E	Chromosome congression, SAC	+/−	Lung and/or spleen tumors in 20 % of 19-21month mice but decreased incidence of liver tumors	DMBA-induced ()	p19/ARF^−/−^ (); Mad2^+/−^ ()	[46, 59]
Fzr1 (Cdh1)	APC/C cofactor	+/−	Mammary gland and other epithelial tumors in 25 % 20-30month mice	DMBA/TPA-induced skin carcinomas ()	NT	[42]
Cdc20	APC/C cofactor	+/AAA (***)	Hepatomas and lymphomas in 50 % of 24 month mice	NT	p53^−/−^ (); Atm^−/−^ ()	[43, 125]
Pttg1	Securin, prevents chromatid separation	−/−	Testicular and splenic hypoplasia, thymic hyperplasia	NT	Rb^+/−^ ()	[123, 136]
		overexp. (transgenic mouse expressing human securin in pituitary cells)	Hyperplasia and microadenomas	NT	Rb^+/−^ (anterior lobe; intermediate lobe =)	[137, 138]

Lck-Cre: a Cre recombinase expressed under the control of the Lck (lymphocyte protein tyrosine kinase) promoter, promoting excision in a thymocyte-specific manner; (*) H: hypomorphic allele; (**) DK: kinetochore binding mutant; (***) AAA: Mad2 binding mutant; NT: not tested; () increased tumor formation; () decreased tumor formation; (=) no changes in tumor formation

**Fig. 2:**
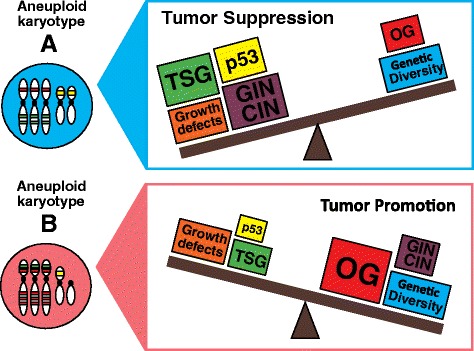
The interplay between the pro- and anti-tumorigenic effects of aneuploidy determines whether cancer is suppressed or promoted. Shown here is a simplistic view of two hypothetical karyotypes and the factors that may come into play to determine their tumorigenic potential

However, many of the reported mouse tumor phenotypes showed incomplete penetrance and typically emerged after a long latency or required carcinogens to emerge (Table 1). Indeed while only ~20 % of CENP-E^+/−^ mice develop tumors in either the spleen or lung at 18–20 months of age [46], BubR1^+/−^ mice do so only when treated with AOM [53]. How can we reconcile these observations that some CIN mouse models are capable of developing spontaneous tumors albeit with long latency but others have no effect on cancer onset or need to be induced by carcinogens? On the one hand, it is conceivable that only specific aneuploid karyotypes favor tumorigenesis or that additional oncogenic mutations (such as presence of oncogenes or inactivation of tumor-suppressor genes) are needed for transformation (discussed below, Fig. 2). This may indicate that the acquisition of a “jackpot” tumor-promoting karyotype would be stochastic, potentially explaining the long latency, low penetrance and low frequency of the onset of spontaneous tumors. Alternatively, the observed differences in cancer susceptibility could be due to different levels of CIN in the various mouse models. Indeed, the levels of CIN present in these different mouse models has been poorly characterized due to the technical difficulty of visualizing chromosomes in mouse solid tissues. Various tissues within each mouse mutant could accumulate different levels of CIN, explaining why certain tissue types are more prone to transformation than others. Another possibility stems from the observation that SAC genes have other non-mitotic functions, making it difficult to disentangle which function is associated to increased cancer susceptibility. Indeed, Mad1 may play a role also in nuclear transport while Mad2 may be involved in the DNA replication checkpoint in yeast [54, 55]. Moreover, BubR1 has been shown to participate in various processes including the DNA damage response and aging, whereas Bub3 can contribute to transcriptional repression during interphase [56–58]. Lastly, while moderate levels of CIN and genome instability could support cancer evolution [15], too much of it might actually hinder the process. From this angle, the observation that some mouse models show decreased tumor onset when challenged with carcinogens could be explained by an exacerbation of CIN driven by drug administration [46, 59] (see below for further discussion).

Aneuploidy is a consequence of CIN and the degree of CIN frequently correlates with karyotypic complexity [60]. However, since cancer genomes are highly complex and contain additional mutations besides chromosome copy number changes, it remains controversial whether aneuploidy acts as a driving force or as a foe of tumorigenesis. An argument supporting the latter hypothesis is that while cancer is a disease of uncontrolled cellular proliferation, aneuploidy has been demonstrated to have deleterious effects on cellular fitness in both yeast and mammalian cells [61–63]. For example, trisomic mouse embryonic fibroblasts (MEFs) displayed decreased growth and resistance to immortalization in respect to their euploid counterparts [64]. However, analysis of proliferation is typically assayed under optimal growth conditions where euploid cells are at the peak of their fitness. It is important to note that cellular fitness is context-dependent and that the aneuploidy-driven phenotypic variation could allow for adaptation to stressful or harsh environments, conditions where euploid cells are not well adapted [62]. In accordance, several observations have been reported where, in challenging environments, aneuploid eukaryotic cells hold fitness advantages in respect to their euploid counterparts [25, 62, 65]. Since this topic has been recently reviewed [62, 66, 67], here we will only briefly describe a few studies that utilized different model systems. Half of the clinical isolates of the pathogenic yeast *Candida albicans* that are resistant to the antifungal drug fluconazole carry extra copies of chromosome 5 [68]. Interestingly, the drug target, *ERG11*, and a main regulator of drug efflux pumps, *TAC1*, genes are both encoded on chromosome 5 [69], indicating that aneuploidy could provide drug resistance by up-regulating the expression of these genes. In the non-pathogenic yeast *Saccharomyces cerevisiae*, aneuploid strains have been shown to display phenotypic advantages in stressful environments or under genetic challenges [70, 71]. Moreover, in the same model system, it has been recently shown that karyotypic diversity allowed adaptation to cytotoxic compounds [72]. In mammalian cells, human pluripotent stem cells (hPSCs) frequently acquire recurrent karyotypic changes in culture [73]. It has been proposed that these aneuploidies are selected during expansion because they confer increased survival to apoptotic signals and reduced differentiation potential [74]. Indeed, hPSCs trisomic for chromosome 12 were found to have increased rates of replication, enhanced tumorigenicity, and gene expression profiles that were similar to germ cell tumors [75]. Human embryonic cells (HE35) with an extra chromosome 8 displayed decreased proliferation rates but lost contact inhibition [76], suggesting that aneuploidy could provide phenotypic traits typical of transformed cells. These studies provide evidence that while aneuploidy is detrimental in conditions where the euploid state is at its peak of fitness, it could provide selective advantage in harsh environments leading to its selection and fixation in the population. Since cancer cells have to acquire phenotypic traits that make them successful in harsh conditions (such as hypoxia or presence of chemotherapy), aneuploidy could act as a mutation that contributes positively to their success (Fig. 1).

Another argument supporting the “foe hypothesis” stems from the observation that aneuploidy has been associated with defective development and lethality in multicellular organisms [63]. In mice and humans, all autosomal monosomies and almost all trisomies result in embryonic lethality. Only mouse trisomy 19 and human trisomy 13, 18, and 21 (Patau, Edward’s and Down syndrome, respectively) are viable although patients develop numerous developmental defects and premature death [77, 78]. Since aneuploidy profoundly affects the transcriptome [61, 71, 79, 80], these embryonic and organismal defects are most likely caused by deregulation of the transcriptional programs that underlie development. But what is deleterious for multicellular organisms might not be bad for individual cells. Indeed, as discussed above, aneuploidy-induced transcriptional changes might confer selective advantage to cancer cells that escaped cellular homeostasis mechanisms [65].

On the other hand, many observations support the hypothesis that whole-chromosome aneuploidy could serve as a driver of cellular transformation [38, 81–83]. Aneuploidy is frequently found in cancer and about 70 % of all solid tumors are aneuploid [84]. Supporting the idea that aneuploidy is not just a simple byproduct of transformation, recurrent chromosome gains and losses between cancer cells were found when Roschke *et al.* profiled the karyotypes of the NCI-60 panel of cancer cell lines [85]. Collection of cytogenetic analyses from a large dataset also showed recurrent karyotypic patterns in primary tumors [60]. Two of the most recurrent cytogenetic abnormalities observed among different types of cancers were gain of chromosome 8q (encoding the MYC oncogene) and loss of 17p (encoding the TP53 tumor suppressor gene) [60], suggesting that aneuploidy could underlie transformation by amplification of oncogenes or loss of tumor suppressors (Fig. 1). Providing compelling evidence in favor of this hypothesis, a recent study found that the number of tumor suppressor genes and oncogenes encoded on each chromosome predicts the likelihood that a given chromosome is preferentially gained or lost in tumors [86], shedding light on the forces shaping karyotype complexity in cancer.

Thus, the above findings suggest that aneuploidy is capable of promoting tumor progression by allowing direct acquisition of cancer-promoting mutations. However, it is unclear whether aneuploidy alone is sufficient to initiate tumorigenesis. Individuals with constitutional aneuploidies, especially those with Down Syndrome (DS), may give us some clues [87]. DS patients have an elevated risk of childhood leukemia including acute lymphoblastic leukemia (ALL) and acute megakaryoblastic leukemia (AMKL) [88]. Moreover, trisomy 21 is the most common cytogenetic abnormality in non-DS ALL patients [89] and is significantly present in pediatric AMKL [90]. Chromosome 21 harbors two leukemia-related hematopoietic transcription factors, ETS2 and ERG, and it has been shown that extra copies of these two genes induce megakaryopoiesis and may have direct roles in promoting AMKL in DS patients [91, 92]. On top of protein-coding genes, microRNAs encoded by aneuploid chromosomes can also result in widespread changes in gene expression [93]. For example, miR-125b-2 found on chromosome 21 has been implicated in the pathogenesis of trisomy 21-associated AMKL via its role in enhancing proliferation of progenitor cells [94].

At the same time, DS patients were found to have a lower incidence of solid tumors when compared to aged-matched healthy individuals [88, 95]. This protective effect has also been recapitulated in mouse models of DS [96, 97]. Part of the tumor-protective effect has been attributed to the gain of a third copy of the Down syndrome critical region-1 (Dscr1) gene, a calcineurin inhibitor that acts as a suppressor of VEGF-mediated angiogenic signaling [98]. Accordingly, a single extra copy of Dscr1 was enough to suppress tumor vascularization and increase apoptosis of lung tumor cells in a mouse model for trisomy 21 [99]. These examples clearly illustrate the multitude of effects, both oncogenic and tumor suppressive, exerted by the presence of extra chromosomes. This dual outcome might result from the fact that aneuploidy alters expression of many genes at the same time, some of which could promote tumor onset or progression while others could perform inhibitory roles. We thus predict that the final outcome on tumor progression caused by an extra chromosome depends on the net effect of all gene expression changes and how these complex changes interact with the tumor microenvironment or the tumor’s specific growth needs (discussed below, Fig. 2).

Besides trisomy 21, humans with other constitutive aneuploidies are also prone to cancer development (reviewed by [87]). It must be noted that it is difficult to determine cancer incidence for many of these patients due to early death. However, it has been shown that Edward’s syndrome patients (trisomy 18) have increased incidence of Wilm’s tumor and hepatoblastomas, while rare individuals with constitutional trisomy 8 have high risk of myeloid neoplasms (reviewed by [87]). Additionally, Turner syndrome patients (X monosomy) showed an increased risk of CNS tumors, ocular cancer, gonadoblastoma and bladder and urethral cancers, while their risk for breast cancer was instead found to be reduced [100]. Men with Klinefelter syndrome (extra X) were found to have increased incidence of certain cancers including that of the lung, breast and lymphoid cells, while there was a decreased risk of prostate cancer [101].

## Aneuploidy: a novel path toward genomic instability?

Is the effect of aneuploidy on tumorigenesis solely dependent on the direct effects on gene expression or could aneuploidy exert other effects on the cellular phenotype? Is it conceivable that having an abnormal chromosome content may result in increased genomic instability [60, 66]? Sheltzer *et al.* showed that aneuploid budding yeast strains increased genetic and karyotypic instability [102]. Indeed while some of them had reduced capacity of transmitting an artificially introduced chromosome as well as defects in mitotic recombination, other aneuploid yeast strains exhibited increased DNA double-stranded breaks, possibly due to defects in DNA replication [102]. A recent study also showed that aneuploid budding yeast strains enter into mitosis in presence of unrepaired DNA, possibly leading to accumulation of chromosomal translocations [103]. Increased CIN was also found in some aneuploid yeast strains generated by sporulation of triploid or pentaploid yeast [71, 104]. Together these studies show that cells harboring different karyotypes are endowed with different degrees of CIN possibly depending on the identity of the genes encoded on the unbalanced chromosomes (Fig. 1).

Mammalian aneuploid cell lines have been generated using either microcell-mediated chromosome transfer or drug-induced chromosome missegregation [76, 105–108]. However, due to technical challenges in generating cell lines containing specific chromosomes in aneuploidy, analysis of genomic instability has only been performed on a few aneuploid karyotypes. While HE35 cells trisomic for chromosome 8 showed a slight increase in structural chromosomal aberrations [76], human renal carcinoma cells with extra chromosome 3 displayed unbalanced chromosomal translocations due to asynchronous and incomplete DNA replication [105], suggesting that aneuploidy could cause genetic instability also in human cells. Aneuploidy has also been shown to increase chromosome missegregation [109]. Indeed, increased sporadic gains or losses of chromosomes were observed in phytohemagglutinin (PHA)-stimulated lymphocytes of humans with constitutive aneuploidies (trisomic 13, 18, 21 and monosomic X patients) [110, 111], suggesting that aneuploidy increases CIN. However, the fact that different aneuploid cell lines cultured *in vitro* maintained a relatively stable karyotype [106, 107], suggests that not all aneuploid karyotypes induce CIN. Supporting this view, aneuploid primary cell lines generated from aborted fetuses did not show an increase in CIN when assayed by FISH [112] and trisomic 8 HE35 cells did not show an increase in micronuclei formation [76], a hallmark of chromosome segregation defects. The discrepancies between these studies could be attributed to differences in cell types, specific karyotypic effects or sensitivity of assays used to measure CIN (particularly FISH vs. metaphase spreading). It is however tempting to speculate that the aneuploidy-induced genome instability could at least partially account for the increased cancer risk observed in constitutive aneuploid patients [87].

We are only now starting to understand the molecular mechanisms that underlie the increased rate of chromosome missegregation and formation of DNA damage in aneuploid cells [60, 66]. Genome stability relies on the activity of several protein complexes involved in tightly regulated processes such as chromosome alignment and DNA replication. Alteration of the stoichiometry between different subunits of such complexes could alter their activity and lead to genome instability. Indeed, mice carrying heterozygous mutations in the SAC components are prone to chromosome missegregation and accumulation of aneuploid cells (reviewed in [37]). In both budding yeast and mammalian cells, aneuploidy has been shown to induce mRNA and protein changes that are on average proportional with chromosome copy number changes [71, 75, 106, 107, 113]. Therefore, when aneuploidy strikes it could lead to changes in the protein stoichiometry of complexes required for genome maintenance. Supporting this hypothesis, it has been shown in budding yeast that copy number imbalances between chromosome VII and X resulted in changes in the ratio of *MAD1* and *MAD2* mRNA, leading to increased CIN [104], possibly due to altered SAC functionality. Under this perspective, by bringing about specific expression changes, different karyotypes would thus have varying effects on CIN and GIN. Accordingly, a variety of CIN and genetic instability levels were reported for different collections of aneuploid yeast strains carrying different aneuploid chromosomes [102, 104, 71]. Another hypothesis that could explain the increased GIN observed in aneuploid cells is that the presence of an extra chromosome challenges the DNA replication or chromosome segregation capacity of the cells. However, Sheltzer *et al.* did not observe increased GIN in budding yeast carrying exogenous artificial chromosomes [102], suggesting that genome instability is not brought about by the mere presence of extra DNA but instead is the result of imbalances in specific gene products.

Recent observations link chromosome missegregation with the generation of DNA damage (Fig. 1) [108, 114, 115]. Janssen *et al.* recently showed that lagging chromosomes trapped in the spindle midzone are prone to damage by the cleavage furrow during cytokinesis, linking chromosome missegregation to DNA damage, chromosome breaks and translocations [115]. However, other studies have not found significant signs of DNA damage in merotelically attached lagging chromosomes [114, 116]. Instead, Crasta *et al.* reported that missegregated chromosomes which become encapsulated in micronuclei are vulnerable to DNA damage and extensive DNA pulverization due to defects in DNA replication [114]. Since chromosomes in micronuclei are also capable of undergoing normal condensation and successfully rejoin the other chromosomes in the next mitosis [114], any DNA damage generated in the micronuclei can potentially be inherited by the daughter cells. An indirect mechanism linking chromosome missegregation and CIN could be that the presence of lagging chromosomes in the spindle midzone has been reported to lead to cytokinesis failure, cleavage furrow regression and formation of binucleated tetraploid cells [117, 118]. In turn, tetraploid cells are known to form multipolar spindles and to undergo chaotic mitoses leading to CIN [119, 120]. Therefore, not only the state of aneuploidy but also the act of becoming aneuploid (i.e. chromosome missegregation) could directly or indirectly increase genome instability (Fig. 1). To close the loop, it has been recently shown that activating the DNA damage response during mitosis by DNA-damaging drugs or ionizing radiation resulted in increased chromosome missegregation through stabilization of kinetochore-microtubule interactions by Aurora A and PLK1 kinases [121]. It is possible that the DNA damage generated by aneuploidy could result in increased CIN and further DNA damage, which would subsequently generate more aneuploidy, leading to a snowballing effect towards chromosomal chaos and GIN (Fig. 1).

## Aneuploidy, CIN and tumorigenesis: more complex than a ménage a trois

As discussed above, some cancer hallmarks could be acquired by the phenotypic variation brought about by gaining or losing specific chromosomes or by the GIN that is associated with aneuploidy or chromosome missegregation. It follows that aneuploidy and CIN should act as drivers of cellular transformation. However, the relationship between aneuploidy, CIN and tumorigenesis is not straightforward (Fig. 2). While some CIN mouse models develop spontaneous tumors, others do not or even have decreased incidence when challenged with carcinogens or combined with tumor-prone backgrounds (Table 1). Indeed, Silk *et al.* showed that exacerbating the level of CIN in CENP-E^+/−^ mice by crossing them to Mad2^+/−^ or p19^ARF−/−^ mice or by treating them with the chemical carcinogen DMBA resulted in enhanced cell death and reduced tumor incidence [59]. Moreover, while BubR1^+/−^Apc^Min/+^ compound mutant mice had drastically increased numbers of colonic tumors they show a reduction of small intestinal polyps compared to Apc^Min/+^ mice [122]. Similarly, Pttg^+/−^ (Securin) mice have decreased pituitary tumor incidence in the Rb^+/−^ background [123]. Collectively, these observations suggest that a moderately elevated rate of CIN could potentially allow transformation while too much or too little CIN would have no effect or even inhibit the carcinogenesis process [59, 124]. In agreement with this hypothesis, while poor life expectancy has been linked to moderate levels of CIN, high CIN level in cancer cells was associated with better prognosis [15].

How can we explain these observations? While too little CIN would not provide a large enough karyotypic variation thereby limiting the possibility of cells to acquire cancer hallmarks, too much CIN could lead to an excessive burden of detrimental mutations and possibly to the rapid loss of beneficial mutations after their acquisition [62]. A moderate CIN instead would allow a population of cancer cells to acquire standing genetic variation, allowing it to adapt towards challenging or fluctuating environments such as presence of chemotherapeutic compounds. Another possibility is that eukaryotic cells from multicellular organisms have acquired surveillance mechanisms that actively prevent the propagation of highly aneuploid cells. In this case, while too much CIN could activate these protection mechanisms and target the cell to death or arrest, a moderate level of CIN might allow aberrant cells to fly under the radar and to keep proliferating. Accordingly, the tumor suppressor p53 is upregulated upon aneuploidization and has been shown to limit the proliferation of aneuploid cells in culture [116, 125]. Moreover, reducing the levels of p53 in SAC-deficient mice showed increased T cell lymphoma and decreased survival [125–128], suggesting that p53 could limit the tumorigenesis potential of CIN *in vivo* by restraining the viability of aneuploid cells (summarized in Table 1). In agreement, thymic apoptosis observed in Cdc20^+/AAA^ mice, presumably triggered by presence of aneuploid cells, was completely rescued upon depletion of p53 [125]. In Drosophila, CIN induced by SAC mutations was also shown to induce apoptosis that was independent of Dp53 but was abrogated by inhibition of the c-Jun N-terminal kinase (JNK) [129]. This observation suggests that stress pathways could also play a role in restricting the viability of aneuploid cells and is in accordance with the evidence that the stress kinase p38 has also been shown to control the proliferation of human aneuploid cells [116].

What signals might activate p53 or p38 in aneuploid or CIN cells? The DNA damage generated upon chromosome missegregation or aneuploidy (discussed above) might represent one such signal sensed by p53. Furthermore, CIN cells in Cdc20^+/AAA^ mutant mice showed increased reactive oxygen species (ROS) production, leading to oxidative DNA damage and subsequent activation of DNA damage response kinase ATM and p53 [125]. Regardless of the precise molecular details, it is tempting to speculate that depleting p53/p38 levels releases a proliferative block in aneuploid cells. Since aneuploidy itself could start a vicious cycle leading to CIN and GIN (Fig. 1), abrogation of the aneuploid proliferative block could lead to accumulation of even more genomic aberrations fostering cancer evolution. Indeed some mouse models of CIN showed increased or accelerated spontaneous tumor onset when combined with p53 mutations (Table 1). Logically, if p53 was involved in restraining the tumorigenic potential of CIN cells, p53 mutations should precede the appearance of CIN in some tumors. Accordingly, during the neoplastic progression of Barrett’s esophagus, p53 loss via chromosome 17p deletion arises before development of aneuploidy [130]. However, in many other cancers such as colorectal carcinoma, CIN is observed as an early event while p53 inactivation occurs much later in tumor progression [19]. Additionally, many aneuploid or CIN cancer cells still express wild-type p53 (http://cancer.sanger.ac.uk/cancergenome/projects/cell_lines) [131]. Indeed, the fact that cells derived from humans with aneuploid conditions or from some organs of healthy individuals [132, 133] can survive in the presence of wild-type p53, could suggest either that aneuploid cells cannot trigger a p53 response at all or that other still unknown mechanisms may take part in restraining the growth of aneuploid cells. Therefore, while a few studies have started to shed some light on the root of the proliferative block of mammalian aneuploid cells, we are still missing a mechanistic and comprehensive description of the signaling pathways and players that link aneuploidy and CIN to impaired proliferation in multicellular eukaryotic cells.

In summary, we hypothesize that the net tumorigenic capacity of each aneuploid karyotype is a complex sum of various factors including the levels of CIN and GIN induced by the specific chromosomal imbalance, presence of oncogenes and tumor suppressor genes on the gained or lost chromosomes, functionality of aneuploidy-suppressive mechanisms and detrimental effects and level of stress encountered by the cell due to its karyotypic abnormalities (Fig. 2). In a simplistic view of a weighing scale, if the costs outweigh the benefits endowed by the cell’s karyotype, tumor suppression will be the end result. However, if advantages conferred by the change in chromosome copy number are capable of overcoming the fitness tradeoffs exerted by aneuploidy, cancer formation may then be promoted.

## Future directions

In this review, we summarized current literature providing evidence that CIN and aneuploidy could promote cancer evolution and discussed possible direct and indirect mechanisms underlying this phenomenon. Lastly, we highlighted the complexity of this ménage à trois, providing possible explanations on why aneuploidy, CIN, GIN and cancer do not have a linear relationship (Fig. 1) and what could limit the tumorigenic capacity of aneuploid cells (Fig. 2). In the future, given its potential role in promoting tumorigenesis, it will be fundamental to characterize the interplay between aneuploidy and GIN in mammalian cells. Does aneuploidy increase the rate of DNA damage and chromosome missegregation? And if so, what are the molecular mechanisms underlying such phenomenon? Is it the presence of specific chromosomes or it is due to lack of scaling of structures required for genome stability? Moreover, what does limit the proliferation of some aneuploid mammalian cells? Does p53 work alone or are there other players? What are the cellular signals sensed by such mechanisms? Answers to these questions are likely to lead to novel strategies to treat cancer and to curb its evolution towards more aggressive and drug-resistant phenotypes.

## Abbreviations

GIN: Genomic instability
CIN: Chromosomal instability
MVA: Mosaic variegated aneuploidy
SAC: Spindle assembly checkpoint
DMBA: Dimethylbenzanthracene
AOM: Azoxymethane
MEF: Mouse embryonic fibroblast
hPSCs: Human pluripotent stem cells
DS: Down syndrome
ALL: Acute lymphoblastic leukemia
AMKL: Acute megakaryoblastic leukemia
PHA: Phytohemagglutinin
FISH: Fluorescence in-situ hybridization
ROS: Reactive oxygen species
